# The Road User Behaviours of Chinese Adolescents: Data From China and a Comparison With Adolescents in Other Countries

**DOI:** 10.5334/aogh.2452

**Published:** 2019-05-28

**Authors:** Huarong Wang, Mengying Wu, Xuebing Cheng, David C. Schwebel

**Affiliations:** 1 Nantong University, CN; 2 University of Alabama at Birmingham, US

## Abstract

**Objectives:**

Adolescents experience high rates of road traffic injuries and deaths as pedestrians and cyclists. One likely reason for adolescents’ elevated traffic injury risk is their risky behaviour on the road. This study examined Chinese adolescents’ road behaviour using a nationwide survey.

**Methods:**

Across 7 Chinese provinces, 4,794 adolescents completed the Chinese version of the Adolescent Road User Behaviour Questionnaire (ARBQ). Results from t-tests and ANOVAs described the road behaviours of Chinese adolescent subgroups, and meta-analytic techniques and Kendall’s correlation analysis compared adolescent road behaviours between China and other countries (Iran and a high-income country composite).

**Results:**

Replicating previous reports from other countries, male adolescents in China generally reported more risk-taking on the road than females, and adolescents aged 15 years and over behaved in a riskier manner than younger ones. Adolescents in rural China reported more risky road behaviours than those who lived in cities, and adolescents who lived only with grandparents behaved more riskily than those who lived with parents only or with parents and grandparents. Adolescents previously involved in a traffic injury also reported riskier road behaviours. In cross-national comparisons, Chinese adolescents’ scores on unsafe road behaviours were lower, and scores on safe road behaviours were higher, than those in other nations. However, there were also several commonalities in how adolescents across countries ranked the frequency of engaging in specific risky road behaviours.

**Conclusions:**

Gender, age, living environment, and traffic injury experience affect adolescents’ reports of their risky road behaviour. Chinese adolescents reported more cautious behaviour than those in high-income countries and in Iran. Traffic injury interventions for adolescents should consider adolescent development as part of pedestrian safety training; results also have implications for guiding parents on how to supervise adolescents near traffic and on what infrastructure development strategies might best protect adolescents in traffic situations.

## 1. Introduction

Adolescents represent a vulnerable group for road traffic injury (RTI) [1,2]. According to data from the Global Burden of Disease project, 37,000 youths aged 10 to 19 years died in traffic accidents in 2016, and 2,420,000 youths were injured on the road [3]. The burden of RTIs is particularly pronounced in low- and middle-income countries [4]. In China, 14,140 adolescents aged 10 to 19 years died in traffic accidents in 2016, and 1,034,286 adolescents were seriously injured. Almost half (46%) of these victims were pedestrians or cyclists [3]. RTI is now the second leading cause of death among children ages 10 to 14 years old in China, and the leading cause of death for youth ages 15 to 19 years old [5].

Despite the fact that children quickly gain competency in their ability to interact safely with traffic as they grow [6,7,8], adolescents have elevated risk for traffic-related injuries compared to younger children. Several possible explanations for adolescent traffic injury risk have been offered. One contributing factor may be related to exposure and supervision. As children grow into adolescence, they behave with greater independence and may be exposed frequently to traffic situations, often unsupervised, but without fully-developed skills to engage in traffic [9,10]. Second, adolescents have a proclivity toward risk-taking tendencies that may lead to injury because adolescent traffic injuries are correlated with risk behaviours on the road [2,11,12]. Third, adolescent habits may increase their risk of traffic injuries. Adolescents are more likely to walk at night, with peers, while intoxicated, and without supervision compared to younger ones [13,14,15]. Distracted walking, for example with smartphones, may also increase adolescent traffic injury risk [16,17].

To understand and ultimately prevent adolescent traffic injury risk, researchers need well-validated assessment tools that measure individual and cultural differences in adolescent road behaviour. One commonly-used instrument is the Adolescent Road User Behaviour Questionnaire (ARBQ), a self-report questionnaire designed to measure adolescent pedestrian and cyclist road behaviours. Developed by Elliott and Baughan in the United Kingdom (UK), the ARBQ includes 43 items that are divided into 3 factors: unsafe crossing behaviour, with items relating to crossing the road in an unsafe manner; dangerous playing in the road, with items relating to playing on the road; and planned protective behaviours, with items relating to engagement in safe behaviours on the road [18].

The ARBQ has strong psychometric properties to measure adolescent road behaviour in the UK [18], as well as in other high-income countries, including Belgium [12], New Zealand [19], and Spain [20] and in two middle-income countries, China [21] and Iran [11]. While validating the instrument in many of those cultures, along with offering validation data, some studies also report gender, age, and geographical (e.g., rural versus urban living area) differences among ARBQ respondents. For example, male adolescents generally take more risks on the road than females, as shown in ARBQ data from Belgium, Iran, New Zealand, Spain, and UK [11,12,18,19,20]. Similarly, younger adolescents cross the road more safely than older adolescents in Belgium, Iran, Spain, and UK [11,18,19,20].

Previous reports also offer cultural contrasts. As an example, Iranian adolescents report they engage in the risky road behaviour “have to stop quickly or turn back to avoid traffic” frequently (rated 8th most common behaviour among the 43 items in Iran), while adolescents in the four high-income countries (Belgium, New Zealand, Spain, and UK) reported engaging in this behaviour much less often (ranked 23rd, 30th, 19th and 22nd, respectively) [11,12,18,19,20].

Unlike in the other countries, ARBQ validation data from China do not include detailed information about age, gender, residential area effects, or cultural contrasts compared to other nations [21]. Thus, this paper was prepared to extend the existing literature in two ways: (a) describe adolescent road user behaviour among a large sample of adolescents in China and (b) compare the road user behaviour of Chinese adolescents to data from previous reports of adolescent road behaviour in another middle-income country, in Iran, and in high-income countries. We hypothesized we would replicate in China the age-, gender-, and urban versus rural effects that are reported in other countries. Given cultural and road environment differences—traffic in China is generally busier and more chaotic than in high-income countries—we hypothesized self-reported road behaviour among adolescents in China would be generally more cautious than those in high-income countries. We also hypothesized Chinese adolescents would behave similarly to Iranian adolescents given similarities in economic status in Iran and China, although we anticipated some differences might emerge due to cultural and traffic environment differences, including cultural tendencies toward collectivism in China and patterns of strict rule adherence in Iranian culture compared to Chinese culture.

## 2. Method

### 2.1. Participants

Across 7 provinces in China (Anhui, Guangdong, Hunan, Jiangsu, Shanghai, Shandong, Zhejiang), 4,920 students in grades 5 through 9 were recruited from 29 primary and secondary schools. The sample yielded 4,794 valid questionnaires (effective response rate of 97.3%), representing 2,317 (48.33%) males and 2,469 (51.50%) females, plus 8 responses with unknown gender (0.17%). The students included 1,298 fifth-graders (27%; Mean age = 10.53 years, *SD* = 0.64), 1,144 sixth-graders (23.9%; Mean age = 11.50 years, *SD* = 0.72), 771 seventh-graders (16.1%; Mean age = 12.38 years, *SD* = 0.70), 841 eighth-graders (17.5%; Mean age = 13.39 years, *SD* = 0.72), and 734 ninth-graders (15.3%; Mean age = 14.48 years, *SD* = 0.71). An additional 6 participants were from unknown grade levels. The full age range of the students was 10 to 18 years old, with an average age of 12.56 (*SD* = 1.34) years. Details about the participants appear in Table 1.

**Table 1:** Demographic information concerning the participants.


Variables	*N*	%	Variables	*N*	%

***Age group***			***Living area***		
under 11	760	15.85	city	1503	31.35
11–12	2092	43.64	small urban	1133	23.63
13–14	1560	32.54	rural	2138	44.60
15 and over	376	7.84	***Most common means of transportation to school***		
***Gender***			walk	1969	41.07
male	2317	48.33	bicycle	990	20.65
female	2469	51.50	family vehicle/motorcycle	1323	27.60
***Family members in the home***			public transportation	500	10.43
parents only	2459	51.29	***Traffic injury in past 6 months***		
grandparents only	785	16.37	no	4387	91.51
parents and grandparents	1278	26.66	yes	407	8.49
other	272	5.67			


*Note*: Information was unreported for 6 adolescents’ age, 6 adolescents’ family members in the home, 20 adolescents’ living area, and 12 adolescents’ most common means of transportation to school.

All participating school officials agreed to cooperate with the study, and informed consent was obtained from all students. Approval for the research was obtained from the Nantong University Academic Ethics Committee prior to the study. Participants were guaranteed anonymity and confidentiality of their answers. The study took about 15 to 20 minutes for each participant.

### 2.2. Measures

All participants completed the Chinese version of the ARBQ [21], which consists of 42 items evaluating self-reported road user behaviours. The Chinese version of the ARBQ varies from the original English version by omitting one item, “cross the street less than an hour after drinking alcohol,” which was deemed culturally inappropriate/insensitive to ask in China (the Iranian version of the ARBQ also omits this item for cultural reasons). Translation was conducted using standard translation and back-translation procedures.

Like the original ARBQ, the Chinese ARBQ is divided into three factors: unsafe crossing behaviour (e.g., “Cross whether traffic is coming or not, thinking the traffic should stop for you”), dangerous playing on the road (e.g., “Ride on a skateboard or roller-skates/roller-blades on the road”), and planned protective behaviour (e.g., “Keep looking and listening until you get all the way across the road”). The Chinese version of the ARBQ has strong construct and criterion validity. Internal consistency is 0.87 and test-retest reliability 0.82 [21]. Table 4 lists all items in the order in which they appear in the questionnaire. Responses were made on a five-point scale (1 = never to 5 = very often).

Along with completing the ARBQ, all participants were asked to report demographic information (age, grade, gender, living area, family members in the home, and most common means of transportation to school; see Table 1). All adolescents also were asked whether they had been injured on the road as a pedestrian or cyclist in the preceding six months. Three criteria were used to define an injury, with affirmative answers to any of them suggesting a traffic injury had occurred: visiting a professional medical unit to evaluate for an injury; emergency treatment or care from family members, teachers, or peers; or missing more than half a day from school because of an injury [22]. Four hundred seven (8.49%) participants reported a traffic injury in the preceding six months.

### 2.3. Data analysis

Data analysis proceeded in three steps. First, a series of descriptive statistics analyses, *t*-tests, and ANOVAs were used to describe the pedestrian behaviours of Chinese adolescents and to compare behaviors across subgroups. Second, meta-analysis technique was used to aggregate and obtain the means of adolescents’ pedestrian behaviours in four high-income countries—Belgium, New Zealand, Spain, and UK—based on published data from those nations. Third, we compared the road use behaviours of Chinese adolescents to adolescent road behaviours in the high-income countries and in Iran through a comparison of means and rankings using *t*-test and Kendall’s correlation analysis. All analyses were conducted using SPSS 22.

## 3. Results

### 3.1. Adolescent road behaviours in China

Table 2 lists descriptive data from the three ARBQ factors for the full sample of Chinese adolescents, and it is divided by available demographic and descriptive variables.

**Table 2:** Subgroup comparisons among Chinese adolescents between scores on ARBQ factors through *t*-test and ANOVA.


Variables	*N*	Unsafe crossing behaviour *M (SD)*	Playing on the road *M (SD)*	Planned protective behaviour *M (SD)*

**Full sample**	4794	1.62(.47)	1.30(.37)	3.16(.63)
***Gender***				
male	2317	1.65(.49)	1.34(.41)	3.12(.66)
female	2469	1.59(.44)	1.27(.32)	3.20(.61)
*t(4786)*		4.83***	7.18***	–1.39
***Age group (years)***				
under 11	760	1.46(.38)	1.18(.28)	3.29(.58)
11–12	2092	1.53(.43)	1.25(.34)	3.21(.67)
13–14	1560	1.76(.48)	1.40(.39)	3.07(.61)
15 and over	376	1.87(.47)	1.44(.35)	3.00(.51)
*F(3,4787)*		151.78***	93.01***	33.22***
***Living Area***				
city	1503	1.57(.46)	1.22(.30)	3.35(.60)
small urban	1133	1.63(.46)	1.30(.34)	3.19(.59)
rural	2138	1.64(.47)	1.35(.41)	3.02(.65)
*F(2,4758)*		11.04***	72.70***	125.53***
***Most common means of transportation to school***				
walk	1969	1.63(.47)	1.34(.40)	3.02(.64)
bicycle	990	1.69(.49)	1.35(.39)	3.19(.62)
family vehicle/motorcycle	1323	1.52(.41)	1.21(.28)	3.34(.61)
public transportation	500	1.72(.49)	1.33(.35)	3.19(.61)
*F(3,4781)*		37.76***	46.61***	72.42***
***Family members in the home***				
only parents	2458	1.62(.47)	1.29(.36)	3.22(.62)
only grandparents	785	1.69(.49)	1.43(.45)	2.89(.62)
parents and grandparents	1278	1.58(.44)	1.26(.31)	3.24(.62)
others	266	1.63(.45)	1.30(.36)	3.10(.61)
*F(3,4787)*		8.65***	42.98***	63.59***
***Traffic injury in past 6 months***				
no	4387	1.61(.45)	1.29(.34)	3.19(.62)
yes	407	1.79(.56)	1.49(.52)	2.86(.69)
*t(4792)*		–6.55***	–7.44***	9.23***


*** p < 0.001.

Preliminary analyses showed no interaction effect between grade and gender, so we analyzed gender and grade separately. *t*-tests showed male adolescents behaved in riskier ways and played more in roadways than female adolescents (*p* < 0.001). There was no significant gender difference for the “planned protective behaviour” factor.

ANOVA was conducted to determine the differences across adolescents in different age groups. There were significant differences for all three factors (*p* < 0.001; *η*^2^_*p* unsafe road crossing_ = .11; *η*^2^_*p* playing on the road_ = .07; *η*^2^_*p* planned protective behaviour_ = .41). Bonferroni post-hoc analyses indicated adolescents aged 15 and over behaved more unsafely while crossing than younger adolescents (*p* < 0.01). For playing on the road, adolescents aged 15 and over scored higher than the two youngest age groups (under 11 year olds and 11–12 year olds) (*p* < 0.001), but there was no significant difference compared to 13–14 year olds. Adolescents under 11 years reported planned protective behaviour significantly more often than did all other age groups (*p* < 0.05). Thus, we detected an overall trend for less safe road behaviours and fewer protective behaviours as adolescents grew older.

ANOVA also revealed differences across adolescent reports based on where they lived (*p* < 0.001; *η*^2^_*p* unsafe road crossing_ = .02; *η*^2^_*p* playing on the road_ = .03; *η*^2^_*p* planned protective behavior_ = .05). Adolescents who lived in the city reported safer road crossing and less playing on the road than those who lived in small urban and rural areas (*p* < 0.01). They also reported more planned protective behaviour (*p* < 0.001).

Adolescents who traveled to school using different means of transportation reported different road behaviours (*p* < 0.001; *η*^2^_*p* unsafe road crossing_ = .02; *η*^2^_*p* playing on the road_ = .03; *η*^2^_*p* planned protective behavior_ = .04). Bonferroni post-hoc analyses indicated adolescents who travelled to school most often by family vehicle/motorcycle reported the safest crossing behaviours and played least on the road (*p* < 0.001). They also reported the most planned protective behaviours (*p* < 0.001). We also found differences based on which adults lived in the home with adolescents. Adolescents who lived with only grandparents reported much riskier road crossing and more playing on the road than those who lived with only parents, or with both parents and grandparents (*p* < 0.001; *η*^2^_*p* unsafe road crossing_ = .02; *η*^2^_*p* playing on the road_ = .03). They also reported the fewest planned protective behaviours (*p* < 0.001; *η*^2^_*p* planned protective behavior_ = .04).

Finally, adolescents who reported involvement in a traffic injury within the preceding six months reported less safe road crossing behaviour, more playing on the road, and fewer planned protective behaviours (*p* < 0.001) than those who reported no recent traffic injuries.

### 3.2. Comparison of adolescent traffic behaviour between China and other countries

Our last step of data analysis was to compare Chinese adolescents’ scores on the ARBQ with aggregated data from adolescents in high-income countries (Belgium, New Zealand, Spain, and UK) and from another middle-income country, Iran. Previous data were derived from several publications [11,12,18,19,20], and high-income data were merged using meta-analysis techniques that weighted larger sample sizes more heavily in the aggregation.

As shown in Table 3, Chinese adolescents reported fewer unsafe behaviours than their counterparts both in high-income countries and in Iran. They also performed planned protective behaviours more often than their counterparts elsewhere. Given these findings, we compared Chinese adolescents’ mean scores for each ARBQ item with adolescents in other countries. Table 4 lists the means, standard deviations, and rankings (shown in descending order according to the Chinese adolescents’ mean scores). Kendall’s correlation coefficients suggested the Chinese adolescents’ rankings were reasonably consistent with those of adolescents in Iran (*r* = .59, *p* < .01) and the high-income countries (*r* = .60, *p* < .01).

**Table 3:** International comparisons between scores in ARBQ factors.


Factor	China *M (SD)*	Iran *M (SD)*	4 high-income countries *M (SD)*	*t*(China vs Iran)	*t*(China vs 4 high-income countries)

Unsafe road crossing behaviour	1.62(.47)	2.62(.30)	2.43(.35)	–148.61***	–120.34***
Dangerous playing in the road	1.30(.37)	1.77(.28)	1.64(.35)	–87.96***	–63.38***
Planned protective behaviour	3.16(.63)	2.76(.78)	2.80(.84)	43.74***	39.38***


*** p < 0.001.

**Table 4:** ARBQ item rankings, means, and standard deviations from samples in China, Iran, and 4 high-income countries.


No	Item (How often do you…)	China			Iran			4 high-income countries		

		*R*	*M*	*SD*	*R*	*M*	*SD*	*R*	*M*	*SD*

39	Look both ways before crossing	1	4.26	1.19	1	4.21	1.13	1	4.10	1.05
20	Keep looking and listening until you get all the way across the road	2	4.23	1.13	5	3.32	1.4	4	3.30	1.23
22	Check to make sure traffic has stopped before using a pedestrian crossing	3	4.17	1.15	3	3.42	1.34	2	3.64	1.21
16	Cross at a place that is well lit when it is dark	4	3.79	1.36	2	3.5	1.35	16	2.50	1.17
32	Use lights on your bike when it is dark	5	2.73	1.64	21	2.28	1.61	6	2.83	1.17
41	Use a traffic police (lollipop man/lady) where there is one available	5	2.73	1.44	22	2.27	1.36	22	2.28	1.02
36	Walk in single file on roads without pavements	7	2.67	1.43	6	3.06	1.41	10	2.65	1.58
27	Have to stop quickly or turn back to avoid traffic	8	2.53	1.38	8	2.87	1.31	10	2.65	1.27
13	Make traffic slow down or stop to let you cross	9	2.39	1.35	13	2.57	1.36	12	2.63	1.36
25	Not bother walking to a nearby crossing to cross the road	10	2.22	1.3	12	2.58	1.36	3	3.35	1.15
10	Wear a cycle helmet when riding a bike	11	2.17	1.44	31	1.94	1.38	20	2.36	1.41
29	Cross when you cannot see both ways very well (like on a bend or top of hill)	12	2.14	1.19	17	2.38	1.29	19	2.40	1.02
37	Hang around in the road talking to friends	13	2.01	1.16	35	1.66	1.12	26	2.16	1.13
42	Cross from behind a stationary vehicle	14	1.99	1.15	9	2.77	1.27	13	2.61	1.12
1	Forget to look properly because you are talking to friends who are with you	15	1.88	0.98	15	2.45	1.35	7	2.82	1.12
30	Wear reflective clothing when crossing the road	16	1.86	1.08	30	2.05	1.27	37	1.50	0.89
5	Walk facing the traffic when on roads without pavements	16	1.86	1.2	20	2.32	1.28	14	2.60	1.28
35	Wear reflective clothing when out on foot in the dark	16	1.86	1.17	23	2.25	1.37	35	1.66	1.05
31	Think it is OK to cross safely, but a car is coming faster than you thought.	16	1.86	1.01	11	2.59	1.17	23	2.27	1.03
3	Wear bright or reflective clothing when riding a bike in the dark	20	1.74	1.09	18	2.35	1.44	32	1.83	1.25
8	Get part way across the road and then have to run the rest of the way to avoid traffic	21	1.72	0.97	7	2.9	1.38	5	2.87	1.08
17	Run across a road without looking because you are in a hurry	22	1.62	0.88	28	2.13	1.2	26	2.16	1.13
28	Forget to look properly because you are thinking about something else	23	1.61	0.87	19	2.33	1.22	17	2.46	1.04
14	Cross without waiting for the ‘green man’	24	1.58	0.88	14	2.46	1.4	15	2.53	1.17
18	Cross between parked cars when there is a safer place to cross nearby	25	1.56	0.87	10	2.68	1.3	8	2.80	1.10
33	See a small gap in traffic and “go for it”	26	1.53	0.86	4	3.35	1.36	9	2.77	1.20
34	Not notice a car pulling out (say from a driveway) and walk in front of it.	27	1.48	0.81	16	2.43	1.11	23	2.27	1.05
40	Walking on the road rather than on the pavement	27	1.48	0.84	25	2.21	1.3	21	2.29	1.01
24	Not notice an approaching car when playing games in the road	29	1.44	0.81	33	1.69	1.11	31	1.91	1.07
7	Cross whether traffic is coming or not, thinking the traffic should stop for you	30	1.41	0.78	29	2.09	1.28	25	2.26	1.20
11	Not look because you cannot hear any traffic around	31	1.39	0.73	27	2.14	1.31	17	2.46	1.21
12	Use a mobile phone and forget to look properly	32	1.37	0.77	24	2.24	1.21	29	2.04	1.12
4	Run around in a road (e.g. when playing foot ball or bull dog)	33	1.36	0.75	36	1.58	1.09	28	2.07	1.18
15	Climb over barriers or railings that separate the road from the pavement	34	1.32	0.69	26	2.21	1.18	30	1.95	1.14
23	Ride on a skateboard (or roller-skates/roller-blades) on the road	35	1.30	0.72	32	1.71	1.17	34	1.78	1.15
26	Run into the road to get a ball, without checking for traffic.	36	1.27	0.65	38	1.56	1	33	1.82	1.01
38	Deliberately run across the road without looking, for a dare	37	1.21	0.62	34	1.69	1.04	40	1.37	0.83
6	Ride out into the road on a skateboard without thinking to check for traffic	38	1.21	0.61	37	1.57	1.11	38	1.41	0.84
21	Hold on to a moving vehicle when riding a bike	39	1.20	0.68	39	1.52	1.04	39	1.39	0.89
2	Hold on to a moving vehicle when riding a skateboard/roller-skates/rollerblades.	40	1.14	0.49	40	1.5	1.1	41	1.33	0.82
9	Play “chicken” by deliberately running out in front of traffic	41	1.12	0.47	41	1.5	1.03	43	1.30	0.79
19	Play ‘chicken’ by lying down in the road and waiting for cars to come along	42	1.08	0.43	42	1.5	1.04	42	1.31	0.83
	Cross less than an hour after drinking alcohol	—	—	—	—	—	—	35	1.66	1.12


As shown in Table 4, the most frequently followed adolescent safe road behaviour across all countries was “looking both ways before crossing”. In fact, the top three most common road behaviours among Chinese adolescents focused on observing traffic, and these items were also ranked high in other countries. There also were commonalities across countries in ranking dangerous behaviours, such as “play ‘chicken’ by lying down in the road and waiting for cars to come along” (ranked 40–42 in all three locations), “play ‘chicken’ by deliberately running out in front of traffic” (ranked 41–43), and “hold onto a moving vehicle when riding a skateboard/roller-skates/rollerblades” (ranked 41–43).

However, Chinese adolescents differed greatly from adolescents in both high-income countries and Iran on four items. On one item, Chinese adolescents reported safer behaviours than their counterparts in other countries: Chinese adolescents reported using traffic police (lollipop man/lady) more often to cross roads than their counterparts in other countries. On the other three items, Chinese adolescents reported less safe behaviours. These items all concerned risky behavior while crossing a street: “Get partway across the road and then have to run the rest of the way to avoid traffic”, “cross between parked cars when there is a safer place to cross nearby”, and “see a small gap in traffic and ‘go for it’”.

Chinese adolescents differed from adolescents in high-income countries but not Iran on two items: “wear reflective clothing when crossing the road” and “wear reflective clothing when out on foot in the dark”. In both cases, Chinese adolescents were more safe: they reported wearing reflective clothing more often than adolescents in high-income countries. Finally, Chinese adolescents differed from Iranian adolescents but not high-income countries on three items. Chinese adolescents were less safe than Iranian adolescents on one of those: “hanging around in the road talking to friends”. For the other two items, both of which concerned bicycle safety (using lights at dark and wearing a helmet), Chinese adolescents were more safe than their Iranian counterparts.

## 4. Discussion

The present study had two primary objectives: to investigate road behaviour among Chinese adolescents and to compare Chinese adolescent road behaviour with that of adolescents in high-income countries and in Iran. Our findings corroborated the hypothesized age- and gender-effects, revealing that male adolescents in China behaved in riskier ways and played more on the road than female adolescents, and that adolescents aged 15 years and over reported higher levels of unsafe crossing and dangerous playing in the road, but fewer protective behaviours than younger ones. We also found several other factors influenced Chinese adolescents’ behavior on the road. Adolescents who lived in rural areas reported less safe behaviour on the road than those in urban areas. Adolescents who traveled to school most often by family vehicle or motorcycle behaved less riskily and reported more protective behaviours than those who walked, cycled, or traveled by public transportation to school. Further, our results showed that adolescents who lived only with grandparents reported less safe crossing, more dangerous playing in the road, and fewer planned protective behaviours than adolescents who lived with parents only or with both parents and grandparents. Last, adolescents who had recently been involved in a traffic injury reported more risky behaviour on the road.

Our analysis of cultural differences discovered that Chinese adolescents reported safer road behaviours and more planned protective behaviours than their counterparts in high-income countries and in Iran, although we also detected many similarities across the cultures. Details and explanations for the findings are discussed below.

### 4.1. Gender and age effects on Chinese adolescents’ road behaviours

Consistent with previous studies [11,12,18,19,20], our results suggested that male adolescents reported more unsafe road behaviours than female adolescents. This finding supports epidemiological findings suggesting males have a higher risk of experiencing severe pedestrian injuries than females [23,24]. Explanations for gender disparities in adolescent pedestrian injury rates are diverse and include increased exposure to traffic, higher risk-taking, and impulsive behaviours among male youth and greater compliance to traffic rules among females [25,26].

Our study also showed significant age differences. As reported in some but not all previous research from other cultures, older Chinese adolescents reported more unsafe road behaviours and less protective behaviours than younger ones [11,18,20]. These results, which seem contrary to developmental expectations, imply adolescents may ignore traffic regulations as they grow older. Such non-compliance may be due to decreased supervision around traffic and a concomitant desire among adolescents to establish self-identity and identify with peer norms, creating rebelliousness and independence [9,27]. It may also reflect increasing confidence to handle road traffic situations and therefore ignore safety behaviour rules to achieve efficiency. Either way, the result may offer some evidence to explain epidemiological findings suggesting road traffic injury ranks as the leading cause of death in adolescents aged 15 to 17 years [28].

### 4.2. The impact of other factors on Chinese adolescents’ road behaviours

Our results suggest Chinese adolescents who live in rural areas were more likely to take risks on the road and less likely to carry out planned protective behaviours than those who lived in cities and small urban areas. These results contradict other reports, which suggest adolescent risk-taking is most common in large urban areas [11,18]. Different traffic environments across countries may explain the differences. Chinese rural areas tend to have poorer road infrastructure (e.g., no sidewalks or crosswalks) than cities and small urban areas in China, but they are still crowded with heavy and somewhat chaotic traffic patterns that are not present in rural areas of many other countries. Thus, results from China are logical: adolescents may be forced to take risks in rural China given the road infrastructure they face that is different from the context in rural areas elsewhere.

We also found that Chinese adolescents who lived only with their grandparents reported more unsafe behaviours on the road than those lived with parents only or with parents and grandparents. Given Chinese cultural patterns where rural families often send parents to work in large cities and leave children/adolescents home with grandparents, adolescents who lived only with grandparents were more likely to live in rural areas (72.3% in our sample). In those rural areas, the adolescents faced poor road infrastructure and also may have experienced less intensive supervision from aging grandparents with limited financial means [29,30]. This combination of factors may have led to greater risk-taking among adolescents living with only grandparents in the home.

We found adolescents who travelled most often to school by family vehicle/motorcycle behaved more safely on the road and performed more planned protective behaviours. Those adolescents likely had less exposure to walking on roadways so may have behaved more cautiously. They may also have had more opportunity to receive safety training from their parents, who accompanied them daily to school.

Finally, and consistent with previous studies [2,31], we found that adolescents who had recently been involved in a traffic injury behaved more unsafely on the road. Causality is difficult to prove, but their unsafe behavior may have contributed to their traffic injuries.

### 4.3. Comparing Chinese adolescent road behaviour with adolescents in other countries

As hypothesized, Chinese adolescents were generally more cautious on the road than those in Iran and in high-income countries. They reported safer crossing, less dangerous playing on the road, and more planned protective behaviours. Differences may be culturally driven. Compared with Western culture, Chinese culture values collectivism, an approach that greatly values conformity, social obligation, and group harmony [32]. Because of this, Chinese adolescents may conform to road rules more than adolescents in Western cultures. Alternatively, Chinese adolescents may feel social pressure to conform with rules and, therefore, respond to self-report surveys with responses that comply with social norms. The distinction between Chinese and Iranian adolescents is harder to explain. Although Islamic cultures place less emphasis on collectivism than Chinese culture, Iranian culture enforces regulations strictly; therefore, we might have anticipated Iranian adolescents to report compliance with road safety recommendations and regulations in a manner similar to Chinese adolescents. This pattern did not emerge.

Despite overarching trends for greater caution among Chinese adolescents, ranking of ARBQ items generally matched across the cultures we studied. The three most frequently reported items, as well as the three least frequently reported items among Chinese adolescents, were ranked similarly by adolescents in the comparison countries. Combined with previous findings [11,12,18,19,20], we conclude that in many respects, adolescents behave in similar ways cross-culturally.

The differences that did emerge seem to reflect the cultural and road environment differences present across the countries we studied. Chinese adolescents wore reflective clothing when crossing the road or walking in the dark more frequently than adolescents in high-income countries, for example, perhaps due to differences in public lighting (poor in rural China) and school start/end times (Chinese secondary school students often have required evening sessions, plus darkness occurs at unusual times in parts of China, which uses just one time zone for the entire country) [33].

We also found that Chinese adolescents reported “hanging around in the road talking to friends” more frequently than adolescents in Iran. This may be due to tendencies toward collectivist culture in China, which encourages interdependence, shared activity, and reluctance to be isolated, even when crossing the road [34]. Moreover, our results showed Chinese adolescents reported wearing a helmet more often when riding a bicycle than adolescents in Iran. This result may be related to Islamic culture in Iran: many Iranian females wear a *Chador* when they go out, and this type of clothing makes wearing a helmet difficult while cycling [11].

### 4.4. Implications and limitations

Our results have multiple implications for adolescent road safety, both in China and beyond. First, the results reinforce the fact that pedestrian safety training should be integrated with developmentally driven psychosocial characteristics of adolescents as they grow older. Adolescents quickly gain basic risk perception and safe route planning on the road as they age [9,35], but our results suggest that Chinese adolescents take more risks as they age. Pedestrian safety training must guide youth to safe road behaviour through theory-driven strategies, such as formation of healthy peer norms [36,37].

Our results also have implications for parenting. Chinese parents should continue to supervise their adolescent children’s road behaviours, reminding them of safety guidelines and modelling safe behaviours themselves. Finally, our results have implications for policy, especially with regards to transportation infrastructure in rural China. Adolescents in rural areas of China reported more risky behaviours on the road, perhaps because the transportation infrastructure in rural China is poor. Roads lack sidewalks and crosswalks, for example, and are poorly lit. With improved infrastructure, adolescent pedestrian safety in rural China may improve; future research would be valuable to evaluate this hypothesis.

Although we conducted this investigation with scientific rigour, our study suffered from limitations. First, we relied on self-reports and may have encountered social desirability bias among our participants. We assured research participants we would keep research findings confidential and anonymous, but adolescents still may have exaggerated or altered their responses to appear safer or to comply with societal expectations [38]. Our data may also have suffered from recall bias, as adolescents may have remembered the frequency of particular behaviours on the road and injury experiences over the preceding six months inaccurately. Second, we recruited adolescents from 29 schools across different areas of China, but we failed to include adolescents from some areas, including the west of China, who might display different road behaviour patterns. Third, our sample was overbalanced to younger adolescents and included few adolescents ages 16 or older. Future research might consider more carefully the behaviours of older adolescents as they approach young adulthood.

## 5. Conclusion

Our study found that male adolescents in China engaged in riskier road behaviours and less safe behaviours than female adolescents; adolescents’ unsafe crossing and risky playing increased with age, while planned protective behaviours decreased with age; adolescents who lived in rural areas and those who lived only with grandparents reported more frequent unsafe behaviours; and adolescents who recently experienced traffic-related injuries reported to behave less safely on the road than those who did not experience a recent injury. Compared to adolescents in other countries, Chinese adolescents behaved more safely on the road, although there were many parallels in behaviour across countries.
